# Zinc finger protein 184 prevents α-synuclein preformed fibril-mediated neurodegeneration through the interleukin enhancer binding factor 3-microRNA-7 pathway

**DOI:** 10.1371/journal.pone.0323279

**Published:** 2025-05-07

**Authors:** Jihye Kim, Soojeong Park, Jee-Ho Lee, Ji-Yeong Lee, Joo-Ho Shin

**Affiliations:** 1 Department of Pharmacology, Sungkyunkwan University School of Medicine, Suwon, South Korea; 2 Single Cell Network Research Center, Sungkyunkwan University School of Medicine, Suwon, South Korea; 3 Samsung Biomedical Research Institute, Samsung Medical Center, Seoul, South Korea; Louisiana State University Health Sciences Center, UNITED STATES OF AMERICA

## Abstract

Parkinson’s disease (PD) is a neurodegenerative disorder characterized by a loss of dopaminergic neurons. Recent studies suggested the association of zinc finger protein 184 (ZNF184) with PD. However, the functional role of ZNF184 in PD pathogenesis remains unclear. Therefore, we aimed to confirm this association and the effects of ZNF184 in a mouse model of PD and human patients with PD. We found that ZNF184 levels were decreased in the substantia nigra (SN) of α-synuclein preformed fibril (α-syn PFF)-injected mice and cells treated with PD toxins. Furthermore, ZNF184 was reduced in the cortex and SN of patients with PD, suggesting an association between ZNF184 and PD pathogenesis. In ZNF184-overexpressing cells, RNA-sequencing analysis revealed significant alterations in several protein-coding genes including *interleukin enhancer binding factor 3* (*ILF3*). Bioinformatic analysis identified potential ZNF184 binding motifs within the *ILF3* promoter, and ZNF184 occupancy was confirmed. Since ILF3 inhibits the biogenesis of microRNA-7 (miR-7), which regulates α-synuclein aggregation, we administered the miR-7 inducer, scutellarin to α-syn PFF-injected mice, preventing dopaminergic neuron and reinstating motor abilities. Our findings suggest that ZNF184 promotes miR-7 upregulation by suppressing ILF3 transcription, revealing a novel pathway that could serve as a promising therapeutic target for the treatment of PD.

## 1. Introduction

Parkinson’s disease (PD) is the most common neurodegenerative disorder worldwide. Clinically, patients typically manifest motor symptoms such as slow movement, tremors, rigidity, and balance disturbances, in conjunction with other non-motor symptoms such as anxiety, depression, autonomic dysfunction, and cognitive impairment. Despite the availability of effective treatments to manage symptoms, no established neuroprotective therapy currently exists for PD [1].

PD is associated with numerous genes including *SNCA, LRRK2*, *parkin, PINK1,* and *DJ-1*, [2]. We demonstrated that zinc finger protein 746 (ZNF746), also known as parkin-interacting substrate (PARIS), is an authentic substrate of parkin and PINK1 [3,4]. PARIS is modulated by the ubiquitin-proteasome system through interactions with parkin and PINK1, contributing to the regulation of the transcriptional coactivator peroxisome proliferator-activated receptor gamma coactivator 1-alpha (PGC-1α) and its downstream gene nuclear respiratory factor 1 (*NRF-1*) by binding to insulin response elements within the *PGC-1α* promoter [3,4]. PARIS belongs to the Krüppel-associated box (KRAB) zinc finger (K-ZNF) protein family and possesses a KRAB domain at its N-terminus and a C_2_HC/C_2_H_2_ type zinc finger domain at its C- terminus [3]. The K-ZNF protein is an evolutionarily dynamic gene family that plays a critical role in suppressing transposable elements (TEs) in mammalian genomes [5]. In the brain, K-ZNF proteins function as transcriptional regulators by interacting with specific DNA and RNA segments [6]. Therefore, K-ZNF proteins are implicated in various human diseases, are particularly important in brain and neural cells, and are associated with neurological disorders [7].

Recent studies have demonstrated that ZNF184 is linked to early-onset PD (EOPD) in Asian populations [8]. ZNF184, located on human chromosome 6p21.3, is a K-ZNF protein with a KRAB domain and 19 C_2_H_2_ zinc finger motifs [9]. Despite its association with PD, the specific functional role of ZNF184 as a transcription factor in PD pathogenesis remains poorly understood.

*Interleukin enhancer binding factor 3* (*ILF3*), located on human chromosome 19 and mouse chromosome 9 [10], is part of the double-stranded RNA-binding protein (DRBP) family, which is involved in RNA metabolism, including the regulation of microRNA (miRNA) biogenesis. ILF3 acts as a negative regulator of microRNA-7 (miR-7) [11,12] which is critical for preventing pathological α-synuclein (α-syn) accumulation [13].

In this study, we demonstrate that ZNF184 transcriptionally regulates ILF3, subsequently influencing miR-7 levels. This regulation is significant because miR-7 helps prevent α-syn aggregation, a major contributor to the formation of Lewy bodies that drive PD pathology.

## 2. Materials and methods

### 2.1. DNA transfection

Human SH-SY5Y neuroblastoma cells (Korean Cell Line Bank, Seoul, Republic of Korea) were cultured in Dulbecco’s Modified Eagle Medium (DMEM) (Gibco, Grand Island, NY, USA) supplemented with 10% fetal bovine serum (FBS) (Gibco) and 1% penicillin-streptomycin (Gibco). For DNA transfection, Xtreme-gene (Roche Holding AG, Basel, Switzerland) and OPTI-MEM (Gibco, Grand Island, NY, USA) were utilized. Additionally, the siRNA-mediated knock-down experiments used Lipofectamine RNAiMAX (Invitrogen, Waltham, MA, USA) as the transfection reagent. Transfection was performed according to the manufacturer’s instructions. After 24 h, the cells were trypsinized (Gibco, Grand Island, NY, USA), and the lysates were harvested.

### 2.2. Plasmids

The Flag-ZNF184 plasmid was generated by creating a pCR8/GW-ZNF184 plasmid using human ZNF184 cDNA, designed using primers based on National Center for Biotechnology Information (NCBI) data, followed by TA cloning with the pCR8/GW/TOPO TA vector (Invitrogen, Waltham, MA, USA). To confirm that the correct sequence was successfully amplified by PCR, DNA sequencing was performed (Cosmo Genetech, Daejeon, Republic of Korea). Gateway LR cloning was performed using the pEZY Flag vector (Addgene, Watertown, MA, USA). A T-blunt PCR cloning kit (Solgent, Daejeon, Republic of Korea) and LR Clonase II (Invitrogen, Waltham, MA, USA) were used for TA and gateway LR cloning. The GFP-ZNF184 plasmid was created through gateway LR cloning using the pcDNA6.2 emGFP vector (Invitrogen, Waltham, MA, USA). Plasmid constructs for the luciferase assay were developed using the pGL3-basic vector (Promega, Madison, WI, USA). The human *ILF3* promoter sequence was obtained from NCBI, and primers with BamH1 and Sal1 restriction enzyme sites were designed and used to clone the sequence into the pGL3-basic vector. The primers used for cloning are listed in S2 Table. For ZNF184 knockdown, siRNA targeting human ZNF184 was purchased from Bioneer (Daejeon, Republic of Korea).

### 2.3. mRNA extraction and RT-qPCR

Cell lysates were subjected to RNA extraction using TRIzol reagent (Invitrogen, Waltham, MA, USA) and chloroform. The extracted RNA was reverse-transcribed into cDNA using amfiRivert cDNA synthesis platinum master mix (GENEdepot, Baker, TX, USA) according to the manufacturer’s instructions. Subsequently, mRNA expression levels were quantified using a RotorgeneQ system (Qiagen, Hilden, Germany) and 2X kapa SYBR Fast qPCR Master Mix (Kapa Biosystems, Cape Town, South Africa). The primers used for RT-qPCR are listed in S2 Table.

### 2.4. MicroRNA extraction and RT-qPCR

MiRNAs were isolated from total cells or tissues using a miRVana miRNA isolation kit (Invitrogen, Waltham, MA, USA) and then subjected to reverse transcription for first-strand cDNA synthesis using a miRCURY LNA RT Kit (Qiagen, Hilden, Germany). Subsequently, miRNA expression levels were quantified using a RotorgeneQ system (Qiagen, Hilden, Germany) and miRCURY LNA SYBR Green PCR kit (Qiagen, Hilden, Germany) according to the manufacturer’s protocol.

### 2.5. Animals and stereotaxic α-syn PFF injection

Eight-week-old male C57BL/6N mice were purchased from Orient (Seongnam, Republic of Korea). Mouse experiments were approved by the Sungkyunkwan University Institutional Animal Care and Use Committee (IACUC, SKKUIACUC2022‐07‐42‐1) and comply with the guidelines of the Association for Assessment and Accreditation of Laboratory Animal Care. The mice were housed under a 12 h light-dark cycle and fed with a standard commercial diet and water ad libitum. The study is reported in accordance with ARRIVE guidelines.

Recombinant mouse α-syn proteins were purified and diluted into 1 × phosphate-buffered saline (PBS) at a concentration of 2.5 μg/μl and then sonicated to prepare α-syn PFF [14]. Mice were anesthetized using isoflurane (1.5- 2% at a flow rate of 0.4-0.8 liter/min) and a total of 5 μg α-syn PFF was bilaterally injected into the SNpc at stereotaxic coordinates ML, ± 1.2 mm; AP, -3.2 mm; and DV, -4.5 mm. The behavioral experiments were performed after a month of α-syn PFF injection. On the following day, mice were euthanized using isoflurane overdose (5% at a flow rate of 0.4-0.8 liter/min) followed by decapitation and brains were collected for analysis.

### 2.6. Human brain tissues

Under the Material Transfer Agreement (MTA), human brain tissues were sourced from the Banner Sun Health Research Institute Brain and Body Donation Program (BBDP) of Sun City, Arizona for the provision of human PD brain tissue. BBDP operates with approval from institutional review boards (IRBs) and follows ethical guidelines for human tissue donation. Written informed consent for brain donation was obtained from all participants or their legally authorized representatives prior to death, in accordance with the ethical standards of BBDP and the Declaration of Helsinki. BBDP is supported by the National Institute of Neurological Disorders and Stroke (U24 NS072026 National Brain and Tissue Resource for Parkinson’s Disease and Related Disorders), the National Institute on Aging (P30 AG19610 Arizona Alzheimer’s Disease Core Center), the Arizona Department of Health Services (contract 211002, Arizona Alzheimer’s Research Center), the Arizona Biomedical Research Commission (contracts 4001, 0011, 05–901, and 1001 to the Arizona Parkinson’s Disease Consortium), and the Michael J. Fox Foundation for Parkinson’s Research. Human samples were accessed for research purposes in October 2014.

### 2.7. Sample preparation and immunoblot analysis

For immunoblotting analysis, cells or tissues were lysed using radioimmunoprecipitation Assay (RIPA) buffer (Thermo Fisher Scientific, Waltham, MA, USA) supplemented with phosphatase (GENEdepot, Baker, TX, USA) and protease inhibitors (GENEdepot, Baker, TX, USA). The protein concentrations were quantified using bicinchoninic acid (BCA) assay reagent (Thermo Fisher Scientific, Waltham, MA, USA). The lysates were mixed with 2 × Laemmli sample buffer (Bio-Rad, Hercules, CA, USA) and boiled at 97 °C for 15 min. Next, 20 μg of the lysates were loaded onto sodium dodecyl-sulfate polyacrylamide gel electrophoresis (SDS-PAGE) gels with varying percentages, depending on the target protein size of interest. The loaded proteins were subsequently transferred onto a nitrocellulose membrane (Bio-Rad, Hercules, CA, USA) and blocked with 5% skim milk. After blocking, the membrane was washed three times with tris-buffered saline with 0.1% Tween® 20 detergent (TBS-T) and subjected to immunoblotting with the desired antibody. For antibodies used in immunoblotting are rabbit anti-ZNF184 (#26100–1-AP, ProteinTech, Rosemont, IL, USA), rabbit anti-ZNF184 (#orb324804, Biorbyt, Cambridge, UK), rabbit anti-NF90/ILF3 (#19887–1-AP, ProteinTech, Rosemont, IL, USA), rabbit anti-poly (ADP-ribose) (PAR) (#4336-BPC-100, Gaithersburg, MD, USA), mouse anti-GFP (sc-9996, Santa Cruz, Dallas, TX, USA), mouse anti-Flag (#A8592, Sigma Aldrich, St. Louis, MO, USA), rabbit anti-TH (#NB300–109, Novus biologicals, Centennial, CO, USA), rabbit anti-α-syn (phospho S129) (#ab51253, Abcam, Waltham, MA, USA), mouse anti-α-syn (#BD-610787, BD bioscience, Franklin Lakes, NJ, USA), and horseradish peroxidase (HRP) anti-β-actin (#ab49900, Abcam, Waltham, MA, USA). Details of the antibodies used are listed in S1 Table. Enhanced chemiluminescence ECL western blotting substrate was utilized for detection (Thermo Fisher Scientific, Waltham, MA, USA).

### 2.8. Subcellular fractionation

SH-SY5Y cells were fractionated into cytoplasmic, membrane, and nuclear components using the subcellular fractionation kit (Thermo Fisher Scientific, Waltham, MA, USA) following the manufacturer’s instructions. The antibodies used in the subcellular fractions were rabbit anti-Hsp90 (#ab13495, Abcam, Waltham, MA, USA) for the cytosolic marker, rabbit anti-Na^+^/K^+^ ATPase (#MA542645, Thermo Fisher Scientific, Waltham, MA, USA) for the membrane marker, and rabbit anti-KDM1/LSD1 (#ab129195, Abcam, Waltham, MA, USA) for the nuclear marker. Details of the antibodies used are listed in S1 Table.

### 2.9. Scutellarin administration

Scutellarin (Sigma-Aldrich, St. Louis, MO, USA) was dissolved in dimethyl sulfoxide (DMSO) (Sigma-Aldrich, St. Louis, MO, USA). Scutellarin was administered to SH-SY5Y cells at a final concentration of 5 μM for 24 h. For *in vivo* experiments, scutellarin was dissolved in drinking water at a concentration of 0.12 mmol/kg and administered for 4 weeks.

### 2.10. Immunohistochemistry

The mice were anesthetized using isoflurane and subsequently perfused with PBS. Following fixation with 4% PFA at 4 °C for 24 h, the brains were incubated in 30% sucrose at 4 °C for 3–4 days. Subsequently, coronal sectioning was performed at 35-mm intervals using a microtome (Thermo Fisher Scientific, Waltham, MA, USA). The sectioned tissues were blocked with a 1 × PBS buffer containing 5% goat serum, 0.1% Triton-X. Subsequently, rabbit anti-TH antibody (#NB300–109, Novus Biologicals, Centennial, CO, USA) was used as the primary antibody, followed by treatment with rabbit anti-biotin antibody (#111-065-045, Jackson ImmunoResearch, West Grove, PA, USA) as the secondary antibody. Both antibodies were added to the blocking buffer. The antibodies used for immunohistochemistry are listed in S1 Table. Subsequently, a Vecstain ABC-HRP kit (Vector Laboratories, Newark, CA, USA) was used to visualize TH-positive neurons in the SN. The tissue was then mounted on glass slides using Distyrene, a plasticizer, and xylene (DPX) mountant (Sigma-Aldrich, St. Louis, MO, USA). Cresyl violet and formalin acetic solutions were used for staining and destaining. Finally, images were taken using a microscope (Leica, Wetzlar, Germany).

### 2.11. Immunofluorescence

Sectioned mouse tissues were permeabilized using 0.1% Triton-X in 1 × PBS for 10 min at room temperature. Subsequently, the membrane was blocked for 1 h in a solution containing 5% goat serum and 0.1% Triton-X in 1 × PBS. Primary antibodies, rabbit anti-TH antibody (#NB300–109, Novus Biologicals, Centennial, CO, USA) and mouse anti-α-syn (phospho S129) antibody (#MMS-5091, BioLegend, San Diego, CA, USA), were diluted in blocking buffer and incubated overnight at 4 °C. For fluorescent labeling, secondary antibodies, donkey anti-rabbit immunoglobulin G (IgG) heavy and light chain (H&L) (#ab150076, Abcam, Waltham, MA, USA) and donkey anti-mouse IgG H&L (#ab150105, Abcam, Waltham, MA, USA) were applied for 1 h at room temperature. 4’,6-diamidino-2-phenylindole (DAPI) (Sigma-Aldrich, St. Louis, MO, USA) diluted 1000 × in 1 × PBS, was used for nuclear staining. Information on the antibodies used in the immunofluorescence experiments is provided in S1 Table. Images were taken using confocal microscopy (Carl Zeiss, Oberkochen, BW, Germany).

### 2.12. ZNF184 consensus sequence identification

To predict the DNA-binding motif of ZNF184, we used the prediction software DNA-binding Specifications of Cys_2_His_2_ Zinc Finger Proteins (http://zf.princeton.edu/index.php). The ‘predict PWM’ category and the protein sequence of ZNF184 were used to retrieve the number of zinc finger domains and their corresponding zinc finger (ZF) scores. After selecting the prediction model for the expanded linear support vector machine (SVM), we examined the prediction results by selecting all zinc finger domains. The outcome simultaneously provided both the sequence and reverse complement sequence logos, revealing key insights into the primary DNA sequence to which ZNF184 might predominantly attach [15].

### 2.13. Chromatin-Immunoprecipitation (ChIP) assay

The ChIP assay was conducted using a SimpleChIP Enzymatic Chromatin IP Kit (Cell Signaling Technology, Danvers, MA, USA) according to the manufacturer’s instructions. SH-SY5Y cells were transfected with 5 μg/μl pEZy-Flag-ZNF184 and cross-linked using 37% formaldehyde. Subsequently, chromatin was fragmented using micrococcal nucleases and ethylenediaminetetraacetic acid (EDTA). Following fragmentation, ZNF184-bound chromatin was extracted using ChIP-grade protein G agarose beads with HRP-conjugated Flag antibody (Sigma-Aldrich, St. Louis, MO, USA), rabbit IgG antibody as a negative control, and histone antibody as a positive control. The elution was reverse-cross-linked using 5M NaCl and proteinase K. Finally, DNA purification was performed, followed by polymerase chain reaction (PCR) using the appropriate primers to confirm the results. The primers used for the ChIP assay are listed in S2 Table.

### 2.14. Luciferase assay

SH-SY5Y cells were transfected for 48 h using the pGL3-basic vector (Addgene, Watertown, MA, USA), pGL3-human ILF3 promoter (pGL3-hILF3), Flag-ZNF184, pGL3-MT1, pGL3-MT2, and pGL3-MT1/2 in Opti-MEM (Gibco, Grand Island, NY, USA) with the Xtreme-gene (Roche Holding AG, Basel, Switzerland). First, the cells were lysed using a passive lysis buffer, and then luciferin, Stop&Glo substrate, and buffer from a dual-luciferase reporter assay kit (Promega, Madison, WI, USA) were added to the samples. Luciferase activity was measured using the Glomax software (Promega, Madison, WI, USA).

### 2.15. Site-directed mutagenesis

To generate pGL3-hILF3 mutants using site-directed mutagenesis, primers were designed using the Agilent QuikChange® primer design program. The sequences are listed in S2 Table. PCR was performed using 2 ng/μl of the pGL3-hILF3 promoter plasmid and kapa HiFi HotStart Ready Mix (2×) (Roche Holding AG, Basel, Switzerland). Subsequently, Dpn1 enzyme (New England Biolabs, Ipswich, MA, USA) was used to digest the template DNA and eliminate non-mutated strands. The resulting PCR products were analyzed for DNA size on a 0.8% agarose gel. Successful mutagenesis was confirmed by DNA sequencing (Cosmo Genetech, Daejeon, Republic of Korea).

### 2.16. Pole test

The pole test was conducted as a behavioral experiment to assess the motor abilities of the mice. Initially, the mice were positioned at the top of a vertical pole approximately 60 cm high. The recording began when the mouse initiated a turn, bringing its head toward the ground. The time required for the mouse to descend the vertical pole until all four limbs were in contact with the ground was recorded.

### 2.17. Statistical analysis

For statistical analysis, we performed a one-way analysis of variance (ANOVA) with Tukey’s HSD post-hoc analysis and an unpaired *t-*test in GraphPad Prism (version 8.0.2). Statistical significance levels were defined as follows: *p < 0.05, **p < 0.01, and ***p < 0.001.

## 3. Results

### 3.1. ZNF184 levels are reduced in the presence of PD-related neurotoxins and α-synuclein preformed fibril (α-syn PFF)

**Fig 1:**
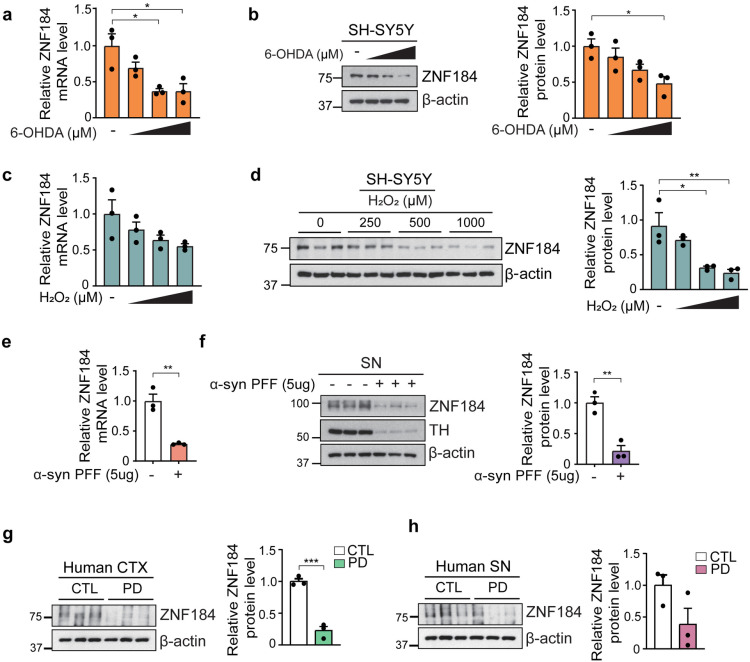
ZNF184 levels are reduced in the presence of PD-related neurotoxins and α-syn PFF. (a) RT-qPCR of ZNF184 mRNA levels and (b) immunoblotting of ZNF184 protein levels with 6-OHDA treatment (0, 25, 50, and 100 μM) in SH-SY5Y cells. (c) RT-qPCR of ZNF184 mRNA level and (d) Immunoblot analysis of ZNF184 protein level with H_2_O_2_ treatment (0, 250, 500 and 1,000 μM) in SH-SY5Y cells. (e) Relative ZNF184 mRNA levels for 5 μg of α-syn PFF administrated to C57BL/6N mice (*n* = 3/group). (f) Immunoblotting of ZNF184 and TH in α-syn PFF administrated to C57BL/6N mice (*n* = 3/group). Quantification data normalized to β-actin. (g, h) Immunoblotting of ZNF184 in CTX (g) and SN (h) samples of a human patient with PD (*n* = 3/group). Statistical significance was assessed through a one-way ANOVA and unpaired *t-*test, with significance levels defined as follows: *p < 0.05, **p < 0.01, and ***p < 0.001.

To evaluate the involvement of ZNF184 in PD pathogenesis, we measured the ZNF184 mRNA and protein levels in the presence of PD-related toxins (Figs 1a–1e). SH-SY5Y cells treated with 6-hydroxydopamine (6-OHDA) or hydrogen peroxide (H_2_O_2_) exhibited a dose-dependent reduction in ZNF184 expression at both the mRNA and protein levels (Figs 1a–1d).

To examine the effects of α-syn PFF on ZNF184 level, we stereotaxically administrated α-syn PFF (5 μg) into the substantia nigra pars compacta (SNpc) of 8-week-old male C57BL/6N mice. After 3 months, the mice were sacrificed, and samples were used for immunoblotting and real-time quantitative PCR (RT-qPCR) analysis. ZNF184 protein and mRNA levels were significantly decreased by > 70% with reduced tyrosine hydroxylase (TH) levels in the substantia nigra (SN) of α-syn PFF-injected mice (Figs 1e and 1f).

Similarly, we observed a significant reduction in ZNF184 protein levels in the cortex (CTX) and a tendency toward a decrease in the SN of human PD patients compared to control subjects (CTL) (Figs 1g and 1h). These findings suggest a strong association between decreased ZNF184 expression and PD pathology.

### 3.2. ZNF184 is a KRAB-zinc finger protein with robust expression in the brain

**Fig 2:**
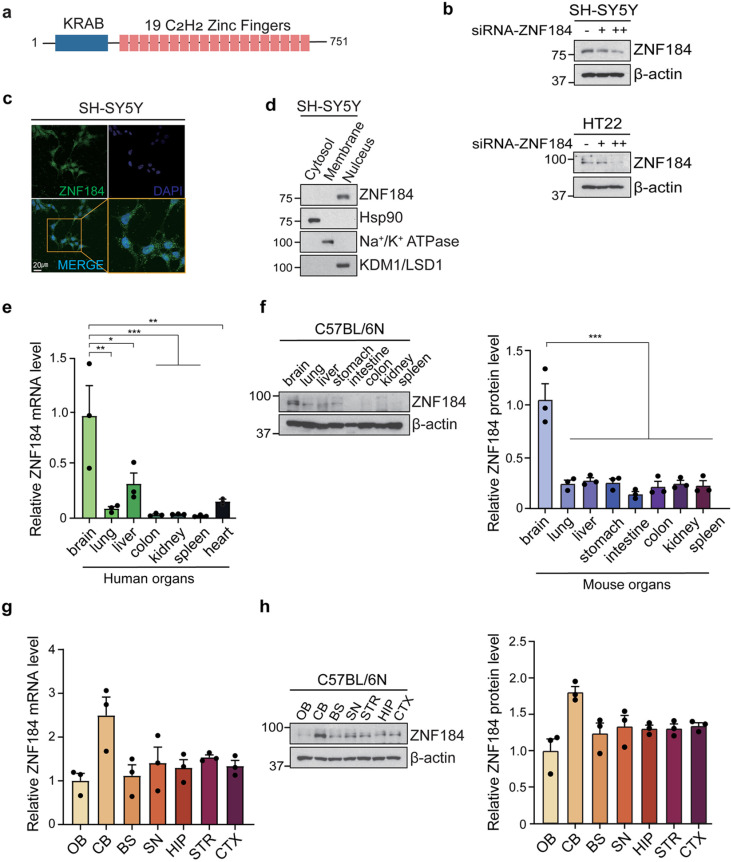
ZNF184 is robustly expressed in the brain. (a) Domain structure of ZNF184 protein with KRAB domain at the N-terminal and 19 C_2_H_2_ zinc finger motif at the C-terminal. (b) Immunoblot analysis of anti-ZNF184 antibody specificity in ZNF184 knockdown SH-SY5Y and mouse hippocampal cell line (HT22) cells. (c) Immunofluorescence images of endogenous ZNF184 in SH-5YSY cells (green: ZNF184, dark blue: DAPI, blue: merge). (d) Subcellular fractionation of SH-SY5Y cells divided into cytosol, membrane, and nucleus compartments indicating the location of ZNF184. Hsp90, Na^+^/K^+^ ATPase, and KDM1/LSD1 were used as subcellular markers for the cytosol, membrane, and nucleus, respectively. (e) Relative ZNF184 mRNA levels in human organs (*n* = 3/group). (f) Immunoblotting and quantification of ZNF184 protein levels in the organs of 3-month-old C57BL/6N mice (*n* = 3/group). (g) RT-qPCR of ZNF184 mRNA levels in various brain regions of 3-month-old C57BL/6N mice (*n* = 3/group). (h) Immunoblotting of ZNF184 protein levels in various brain regions of 3-month-old C57BL/6N mice (*n* = 3/group). Quantification of each data normalized to β-actin. Statistical significance was assessed through a one-way ANOVA, with significance levels defined as follows: *p < 0.05, **p < 0.01, and ***p < 0.001.

ZNF184 is a KRAB-zinc finger protein consisting of 751 amino acids, characterized by a KRAB domain at its N-terminus and 19 C2H2-type zinc finger motifs at its C-terminus [9] (Fig 2a). To confirm the specificity of the ZNF184 antibody, we conducted immunoblot analysis in SH-SY5Y and HT22 cells transfected with siRNA-ZNF184, which validated the effectiveness of the antibody for detecting ZNF184 (Fig 2b).

Immunofluorescence and immunoblotting analyses in SH-SY5Y cells demonstrated that endogenous ZNF184 is distributed in both the nucleus and cytoplasm, with predominant localization in the nucleus (Figs 2c and 2d). Similarly, immunoblot images confirmed ZNF184’s strong presence in the nuclei of SH-SY5Y cells (Fig 2d).

Further analysis of ZNF184 mRNA levels in human organs and ZNF184 protein levels in mouse organs revealed high expression in the brain tissues of both species (Figs 2e and 2f). Specifically, ZNF184 showed robust expression in the mouse cerebellum (CB) at both the mRNA and protein levels (Figs 2g and 2h). To ensure reliable data, we used the ZNF184 antibody (#26100–1-AP, ProteinTech), which underwent thorough validation for immunoblot analysis. We confirmed this antibody consistently detected a single immunoreactive band at the predicted molecular weight, demonstrating high specificity. These findings suggest that ZNF184 plays a significant role in the brain, likely contributing to its cellular functions.

### 3.3. RNA-sequencing identifies putative target genes of ZNF184

**Fig 3:**
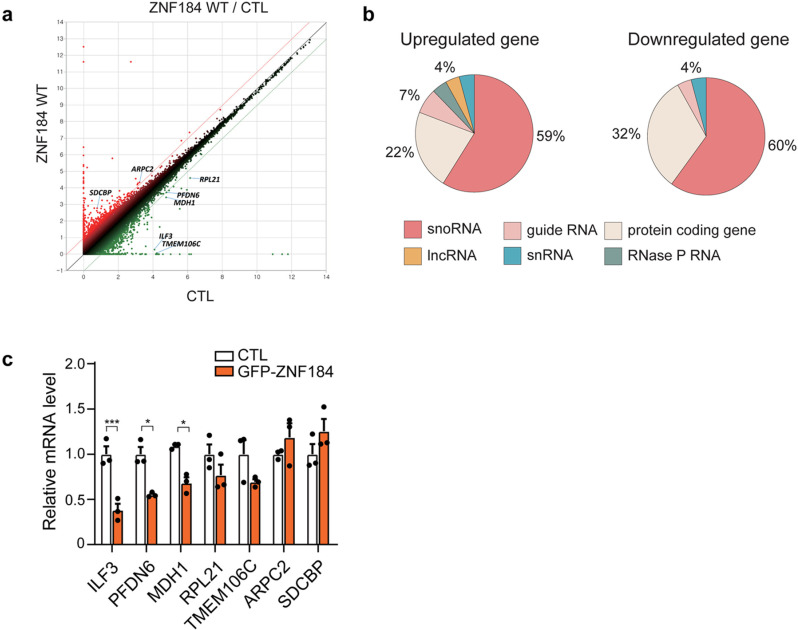
RNA-sequencing identifies the putative target genes of ZNF184. (a) Scatter plot of DEGs in ZNF184-overexpressing HEK293 cells. (b) Pie chart of upregulated and downregulated genes revealed by RNA-sequencing in ZNF184-overexpressing HEK293 cells. (c) Relative mRNA levels of ZNF184 target genes in GFP-tagged ZNF184-overexpressing SH-SY5Y cells. Statistical significance was assessed through Tukey’s HSD post-hoc analysis and two-way ANOVA, with significance levels defined as follows: *p < 0.05 and ***p < 0.001.

RNA-sequencing analysis of HEK293 cells overexpressing ZNF184 revealed substantial changes in various small RNA species and seven protein-coding genes. These genes include *ILF3*, *prefoldin subunit 6* (*PFDN6*), *malate dehydrogenase 1* (*MDH1*), *ribosomal protein L21* (*RPL21*), *transmembrane protein 106C* (*TMEM106C*), *actin related protein 2/3 complex subunit 2* (*ARPC2*), and *syndecan binding protein* (*SDCBP*) (all with p < 0.05 and a fold-change > 2) (Figs 3a and 3b).

To validate these results, we measured the mRNA levels of these seven genes in ZNF184-overexpressing SH-SY5Y cells. The most significant change was observed in *ILF3*, which exhibited a 60% reduction in mRNA levels as confirmed by RT-qPCR (Fig 3c). This finding highlights *ILF3* as a primary target gene regulated by ZNF184, potentially contributing to its downstream effects in cellular processes.

### 3.4. ZNF184 transcriptionally represses ILF3 through occupancy of the *ILF3* promoter

Western blot analysis confirmed that Flag-ZNF184 was successfully overexpressed in HEK293 and SH-SY5Y cells, with exogenous ZNF184 levels approximately three-fold higher than endogenous levels (S1a Fig.). To verify transfection efficiency in SH-SY5Y cells, GFP fluorescence and anti-ZNF184 immunostaining confirmed robust GFP-ZNF184 expression (S1b Fig.), demonstrating the reliability of lipofectamine-mediated transfection.

**Fig 4:**
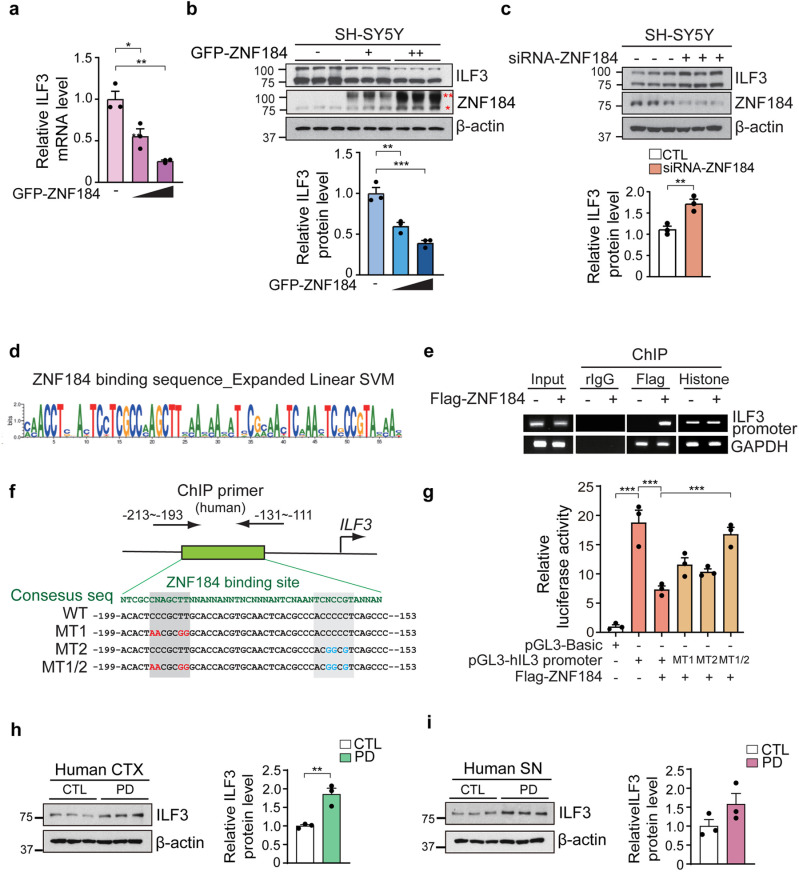
ZNF184 transcriptionally represses ILF3 through occupancy of the *ILF3* promoter. (a) RT-qPCR of ILF3 mRNA levels in SH-SY5Y cells overexpressing GFP-ZNF184. (b) Immunoblot analysis of ILF3 protein levels in the GFP-ZNF184-overexpressing SH-SY5Y cells. ZNF184 antibody detects both endogenous ZNF184 (indicated by a single red asterisk) and exogenous GFP-tagged ZNF184 (indicated by two red asterisks). Quantification of ILF3 protein levels is normalized to β-actin. (c) Immunoblotting of ILF3 and ZNF184 in ZNF184 knockdown SH-SY5Y cells by siRNA-ZNF184. Quantification of the immunoblot normalized to β-actin. (d) Prediction of ZNF184 consensus sequence by expanding linear SVM. (e) ChIP assay of Flag-tagged ZNF184-overexpressing SH-SY5Y cells. Rabbit IgG and histone antibodies are used as negative and positive controls, respectively. (f) Localization of ZNF184 ChIP primer binding site in the human *ILF3* promoter with ZNF184 consensus sequence, human *ILF3* promoter (WT) sequence, MT1 (mutant 1), MT2 (mutant 2), and MT1/2 (mutant 1 and 2) sequences. (g) Luciferase assay of overexpressing SH-SY5Y cells with pGL3-basic vector, pGL3-hILF3 promoter (WT), mutants (MT1, MT2, and MT1/2) and Flag- ZNF184. (h, i) Immunoblotting of ILF3 in CTX (h) and SN (i) samples of a human patient with PD (*n* = 3/group). Statistical significance was assessed through a one-way ANOVA and unpaired *t-*test, with significance levels defined as follows: *p < 0.05, **p < 0.01, and ***p < 0.001.

To investigate whether ZNF184 regulates ILF3 expression, we overexpressed GFP-tagged ZNF184 in SH-SY5Y cells and observed a dose-dependent reduction in ILF3 protein and mRNA levels (Figs 4a and 4b). Conversely, siRNA-mediated knockdown of ZNF184 led to a 1.5-fold increase in ILF3 protein levels (Fig 4c), suggesting that ILF3 is a physiological target of ZNF184. A similar result was confirmed in HT22 cells overexpressing siRNA targeting ZNF184 (S2 Fig.).

To identify the specific ZNF184-binding site on the ILF3 promoter, we utilized a zinc finger protein consensus sequence finder (http://zf.princeton.edu/help.php) [15,16]. We identified a potential ZNF184 binding motif and designed primers targeting this region in the human ILF3 promoter (Figs 4d and 4f, top panel). Chromatin immunoprecipitation (ChIP) assays confirmed that ZNF184 directly binds to the ILF3 promoter (Fig 4e).

To further pinpoint the binding site, we generated three mutant ILF3 promoter constructs (MT1, MT2, and MT1/2) (Fig 4f, bottom panel) and co-transfected them into SH-SY5Y cells with Flag-tagged ZNF184. Luciferase assay showed that ZNF184 successfully suppressed the activity of the *ILF3* WT but not the *ILF3* MT constructs (Fig 4g). The complete loss of repression in the MT1/2 construct suggests that ZNF184 binds to the -199 to -153 region of the ILF3 promoter, identified as “ACACTAACGCGGGCACCACGTGCAACTCACGCCCACGGCGTCAGCCC” (Fig 4g). This direct interaction between ZNF184 and the ILF3 promoter indicates that ZNF184 acts as a transcriptional repressor of ILF3.

Since we found reduced ZNF184 in human PD brains (Figs 1g and 1h), we next examined ILF3 protein expression in the PD CTX and SN (Figs 4h and 4i). Western blot analysis revealed an increase in ILF3 protein levels in PD brains, suggesting a potential dysregulation of the ZNF184-ILF3 pathway in PD pathology (Figs 4h and 4i). However, due to the unavailability of RNA samples from PD patients, we were unable to assess miR-7 levels in the same human tissue samples. Despite this limitation, the observed reduction in ILF3 protein provides valuable insights into the role of this pathway in PD.

### 3.5. ILF3 upregulation represses miR-7 expression and removes phosphorylated α-syn (p-α-syn) in SH-SY5Y cells

**Fig 5:**
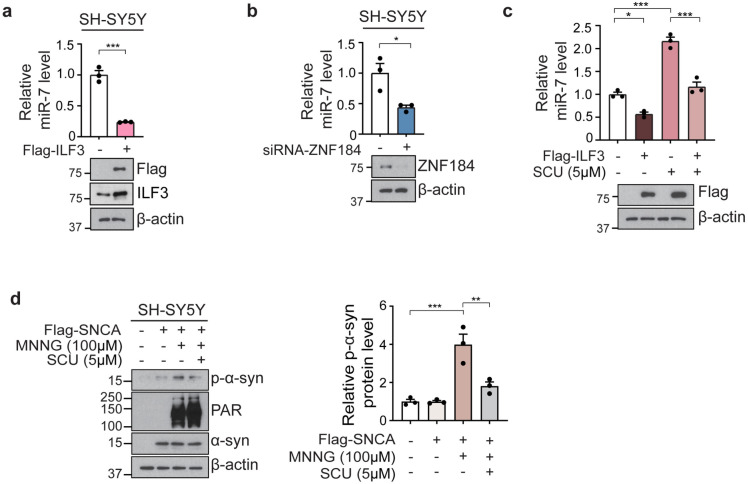
Upregulation of ILF3 represses miR-7 expression and removes p- α-syn in SH-SY5Y cells. (a) Quantification of miR-7 levels in ILF3-overexpressing SH-SY5Y cells, measured by RT-qPCR and normalized to U6 snRNA. Immunoblot analysis was performed using anti-ILF3 and anti-Flag antibodies to detect both endogenous ILF3 and exogenous Flag-tagged ILF3. Immunoblotting confirms successful overexpression of Flag-ILF3 in SH-SY5Y cells. (b) RT-qPCR of miR-7 level in ZNF184 knockdown SH-SY5Y cells by siRNA-ZNF184. Immunoblot analysis shows ZNF184 knockdown in SH-SY5Y cells. (c) RT-qPCR of miR-7 levels in ILF3-overexpressing and scutellarin (SCU)-treated SH-SY5Y cells normalized to U6 snRNA. Immunoblotting confirms Flag-ILF3 overexpression in SH-SY5Y cells. (d) Immunoblotting of p-α-syn in Flag-SNCA-overexpressing SH-SY5Y cells treated with 100 µ M MNNG or with or without 5 µ M SCU. Immunoblot analysis of α-syn and PAR confirms α-syn overexpression and PAR activation in SH-SY5Y cells, respectively. Statistical significance was assessed through a one-way ANOVA and unpaired *t-*test, with significance levels defined as follows: *p < 0.05, **p < 0.01, and ***p < 0.001.

Since ILF3 negatively regulates miR-7 biogenesis [12], we investigated its effect on miR-7 expression by transfecting SH-SY5Y cells with Flag-tagged ILF3. We observed a 76% decrease in miR-7 levels (Fig 5a). Similarly, siRNA-mediated ZNF184 knockdown led to an approximately 55% reduction in miR-7 expression (Fig 5b), indicating that the ZNF184-ILF3 pathway plays a critical role in controlling miR-7 levels.

Given that breviscapine has been reported to upregulate miR-7 [17], we examined whether scutellarin, the primary active ingredient of breviscapine [18] could regulate miR-7 expression. We treated SH-SY5Y cells with 5 μM of scutellarin for 24 h after overexpressing Flag-tagged ILF3 (Fig 5c), demonstrating scutellarin restored the reduction in miR-7 caused by the upregulation of ILF3 to basal levels (Fig 5c). Furthermore, scutellarin treatment alone led to a two-fold increase in miR-7 levels (Fig 5c).

Since miR-7 is involved in the elimination of α-syn aggregation through autophagy [13], we tested whether scutellarin could prevent PAR-mediated α-syn aggregation in SH-SY5Y cells. SH-SY5Y cells were transiently transfected with Flag-tagged α-syn, treated with 5 μM scutellarin for 24 hours, and exposed to 100 μM N-methyl-N’-nitro-N-nitrosoguanidine (MNNG) for 1 hour. The results demonstrated that scutellarin significantly diminished p-α-syn formation (Fig 5d), suggesting a potential therapeutic effect of scutellarin in mitigating α-syn aggregation.

### 3.6. Scutellarin restores dysregulation of the ZNF184-ILF3-miR-7 pathway and prevents α-syn PFF-induced neurotoxicity *in vivo*

**Fig 6:**
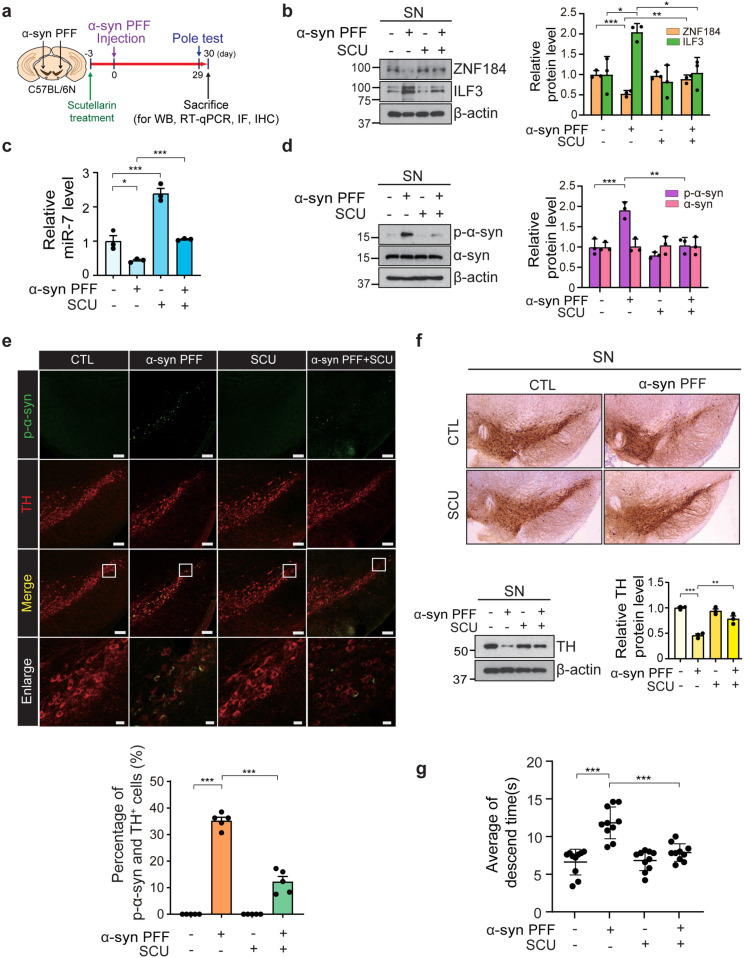
Scutellarin restores the dysregulation of the ZNF184-ILF3-miR-7 pathway caused by α-**syn PFF injection** ***in vivo.*** (a) Schematic illustration of the bilateral injection of α-syn PFF into the SNpc and SCU treatment. (b) Immunoblot analysis of ZNF184 and ILF3 in the 1-month intranigral α-syn PFF injection to the SNpc and SCU treatment. Quantification of ZNF184 and ILF3 protein levels normalized to β-actin. (*n* = 3/group). (c) RT-qPCR of miR-7 normalized to U6 snRNA. (*n* = 3/group). (d) Immunoblots of p-α-syn and α-syn. Relative protein levels of p-α-syn and α-syn normalized to β-actin (*n* = 3/group). (e) Confocal image of immunofluorescence of p-α-syn (green), TH (red), and merge (yellow) in α-syn PFF-injected mouse SN. Scale bar = 100 µm. The scale bar for the enlarged image is 20 µm (*n* = 5/group). Quantification of percentage of p-α-syn/TH (CTL, *n* = 5710 cells for 5 mice; α-syn PFF, *n* = 3992 cells for 5 mice: SCU, *n* = 5849 cells for 5 mice; α-syn PFF + SCU, *n* = 4541 cells for 5 mice). (f) Representative immunohistochemistry image of TH immunostaining of the midbrain section from mouse SN injected with α-syn PFF and SCU treatment. Immunoblot analysis of TH-positive neurons in mouse SN. Quantification of TH protein level normalized to β-actin. (g) Average descent time for α-syn PFF-injected and SCU-treated mice in the pole test (*n* = 10/group). Statistical significance was assessed through a one-way ANOVA, with significance levels defined as follows: *p < 0.05, **p < 0.01, and ***p < 0.001.

To determine whether scutellarin can prevent α-syn PFF-induced dopaminergic neuronal death *in vivo*, we stereotaxically administrated α-syn PFF into the SN of 8-week-old male C57BL/6N. Scutellarin was given orally starting 3 days before α-syn PFF injection. Four weeks after α-syn PFF injection, the mice underwent a behavioral test, following by biochemical and immunohistochemical analysis (Fig 6a).

As shown previously (Fig 1), α-syn PFF injection reduced ZNF184 levels and increased ILF3 levels, resulting in a significant decrease in miR-7 expression in the SN (Figs 6b and 6c). Notably, scutellarin treatment alone elevated miR-7 levels but did not alter ZNF184 or ILF3 expression (Figs 6b and 6c). However, in α-syn PFF-injected mice, scutellarin administration reversed the dysregulation of ZNF184, ILF3, and miR-7 levels (Figs 6b and 6c), and reduced the strong p-α-syn signal induced by α-syn PFF by 50% (Fig 6d). Monomeric α-syn protein levels remained unchanged in all groups (Fig 6d).

Immunofluorescence analysis revealed a robust p-α-syn signal in tyrosine hydroxylase (TH)-positive neurons of α-syn PFF-injected mice, indicating α-syn aggregation, whereas scutellarin significantly reduced the p-α-syn signal (Fig 6e). Furthermore, immunohistochemistry and immunoblotting showed that scutellarin treatment partially rescued the α-syn PFF-mediated dopaminergic neuronal loss, which was approximately 50% in untreated mice (Fig 6f). Behavioral assessment using the pole test indicated that scutellarin administration partially alleviated motor deficits caused by α-syn PFF injection (Fig 6g). These findings suggest that scutellarin effectively reduces p-α-syn accumulation, and mitigates dopaminergic neuronal death and motor dysfunction *in vivo*.

## 4. Discussion

Our study was prompted by the identification of the reported association of ZNF184 with PD [8,19–22]. Although ZNF184 has been investigated in other diseases such as esophageal squamous cell carcinoma (ESCC) [23], its role in PD remains relatively unexplored. Based on the previous studies identifying ZNF184 as a PD-associated gene, we monitored ZNF184 expression under conditions involving neurotoxic PD agents and α-syn PFF injection, which resulted in reduced ZNF184 levels.

Although the mechanism underlying the reduction of ZNF184 expression is not yet fully understood, reactive oxygen species (ROS) might be involved since increased ROS are commonly found in the presence of neurotoxic PD agents and α-syn PFF, triggering the neurodegenerative process in PD pathogenesis [24]. To examine the interaction of ROS-related transcription factors with the *ZNF184* promoter and its expression, we used a bioinformatics tool, Tfsitescan (http://www.ifti.org/cgi-bin/ifti/Tfsitescan.pl) [25], a transcription factor finder, to identify multiple binding sites within the *ZNF184* promoter. These transcription factors, including AP-1, Ets, and C/EBP, are regulated by cellular redox states [26]. Future research is needed to further explore the regulation of ZNF184 by these transcription factors in the context of ROS.

The results of RNA-sequencing analysis to identify the target genes of ZNF184 showed that alterations of a significant proportion of small nucleolar RNAs (snoRNAs) and some protein-coding RNAs in HEK293 cells overexpressing ZNF184. snoRNAs, a type of non-coding RNA, typically act a part in the post-modification and maturation of ribosomal RNA (rRNA). However, many snoRNAs have not yet been assigned a specific function, leading to their classification as orphans [27]. Despite recent research on the roles of orphan snoRNAs, there remains a lack of a comprehensive understanding of their biology [28]. Likewise, despite the increasing acknowledgment of the importance of researching non-coding RNA, we has focused on protein-coding genes because of the essential functional roles of proteins within cells [29] as well as the technical challenges in studying snoRNAs.

In the present study, we focused on ILF3 as a ZNF184 target gene and validated the effects of ZNF184 overexpression and knockdown on ILF3 transcription. To identify the ZNF184 binding sequences on the *ILF3* promoter, we employed a zinc finger protein consensus sequence finder (http://zf.princeton.edu/help.php), which showed that ZNF184 binds to specific sequences in the *ILF3* promoter, thereby transcriptionally regulating ILF3 expression. ChIP assays confirmed that ZNF184 binds to the ILF3 promoter, and a luciferase assay further pinpointed the specific binding site. However, it remains to be determined whether ZNF184 directly interacts with this promoter sequence, requiring further research to clarify the exact nature of this interaction.

ILF3 undergoes alternative splicing, producing two transcripts: ILF3 and NF90 [30]. ILF3 has been studied in relation to cancer [31] and fear memory formation [32], and it plays a key role in RNA metabolism, particularly in the regulation of miR-7 biogenesis [11,12]. MiR-7, a microRNA highly expressed in the brain, is crucial for brain development and protection against neurological disorders, including PD [33]. It protects neurons by activating the mTOR pathway [34] and preventing cell death by targeting proteins like Bax and Sirt2 [35]. Since miR-7 also binds to the 3’ untranslated region (UTR) of α-synuclein mRNA, inhibiting its translation and promoting autophagy-mediated clearance of aggregated α-synuclein [13], we explored the interaction between ILF3 and miR-7 as downstream mediators of ZNF184.

In this study, we used scutellarin, a compound derived from Scutellaria laterifolia, to elevate miR-7 levels. Scutellarin, which is 90% of the active component breviscapine, is known for its therapeutic potential in treating cancer, ischemic stroke, and cardiovascular diseases [18,36]. We found that scutellarin effectively elevated miR-7 levels and reduced phosphorylated α-synuclein (p-α-syn) in SH-SY5Y cells subjected to MNNG-mediated PAR activation and in the substantia nigra of α-syn-PFF-injected mice.

Recent studies reveal that scutellarin binds to α-syn through non-covalent interactions with moderate affinity [37]. Molecular docking analysis indicates that scutellarin interacts with residues in the NAC region and C-terminus of monomeric α-syn, as well as the C-terminal residues of fibrillar α-syn. These interactions appear to inhibit fibrillation by targeting these regions and may contribute to stabilizing the autoinhibitory conformation of α-syn.

Similarly, the anti-amyloidogenic properties of scutellarin have been shown in an Alzheimer’s disease model [38]. Scutellarin was found to destabilize preformed Aβ aggregates and inhibit Aβ aggregation, which improved behavioral impairments and reduced Aβ levels in the APP/PS1 transgenic mouse brain. Additionally, the study demonstrated that scutellarin possesses anti-inflammatory effects by downregulating pro-inflammatory cytokine expression and modulating redox signaling. These effects are attributed to its phenolic structure, which facilitates blood-brain barrier penetration [39,40].

Interestingly, although miR-7 is reported to bind the 3’UTR of α-synuclein and suppress monomeric α-synuclein expression [41], our *in vivo* experiments did not detect significant changes in monomeric α-synuclein levels. Given evidence that miR-7 facilitates the autophagic degradation of aggregated α-synuclein [13], we hypothesize that the observed reduction in p-α-syn in this study may be attributed to enhanced autophagic clearance rather than decreased transcription.

Remarkable studies have further emphasized the significance of post-transcriptional regulation in miR-7 biogenesis. A screening platform, RP-CONA, was developed to identify small molecules, such as quercetin, that disrupt the interactions between HuR and the terminal loop of pri-miR-7–1. This disruption enhances miR-7 maturation and leads to reduced α-synuclein expression, highlighting the therapeutic potential of modulating RNA-protein interactions in PD [42]. Additionally, foundational studies demonstrated that RNA-binding proteins such as HuR and MSI2 regulate miR-7 biogenesis in a tissue-specific manner, either by inhibiting its processing or influencing miRNA-mRNA interactions [43,44]. These findings reinforce the complex regulatory landscape of miR-7 and support our hypothesis that miR-7 may influence α-synuclein aggregation through both transcription-independent and post-transcriptional mechanisms.

Overall, our findings highlight the dual mechanisms of scutellarin in reducing α-synuclein toxicity: promoting miR-7-mediated autophagic degradation and directly interacting with α-synuclein to inhibit fibrillation. Moreover, the observed increase in miR-7 levels, driven by reduced ILF3 and elevated ZNF184, suggests that ZNF184 may hold therapeutic potential for PD prevention and treatment. Future studies are needed to further elucidate the mechanistic role of ZNF184 and its potential as a therapeutic target for PD.

## 5. Conclusions

In summary, we propose that ZNF184 promotes miR-7 upregulation by inhibiting ILF3 transcription, identifying a novel pathway as a potential therapeutic target for PD treatment. Future research should focus on the direct regulatory mechanisms of ZNF184, its interactions with ROS and transcription factors, and its therapeutic application in neurodegenerative diseases.

## Supporting information

S1 Fig Efficient overexpression of ZNF184 in HEK293 and SH-SY5Y cells. (a) Western blot analysis comparing endogenous and exogenous ZNF184 expression levels in HEK293 and SH-SY5Y cells transfected with Flag-ZNF184. (b) Immunostaining of GFP-ZNF184-transfected HEK293 and SH-SY5Y cells. GFP fluorescence (green) and anti-ZNF184 antibody staining (red) confirm robust expression of GFP-ZNF184 in both cell lines. Nuclei were counterstained with DAPI (blue). Scale bar: 25 um.
(TIF)

S2 Fig Immunoblotting of ILF3 and ZNF184 in ZNF184 knockdown HT22 cells by siRNA-ZNF184. Quantification of the immunoblot normalized to β-actin. Statistical significance was assessed through a one-way ANOVA and unpaired *t-*test, with significance levels defined as follows: **p < 0.01.
(TIF)

S1 Table Antibodies used in this study. (DOCX)

S2 Table Primers used in this study. (DOCX)
