# Supplementary figures and images for: Zinc finger protein 184 prevents α-synuclein preformed fibril-mediated neurodegeneration through the interleukin enhancer binding factor 3-microRNA-7 pathway
Source: PLoS One. 2025 May 7;20(5):e0323279. doi: 10.1371/journal.pone.0323279 (PMC12057860; doi:10.1371/journal.pone.0323279)

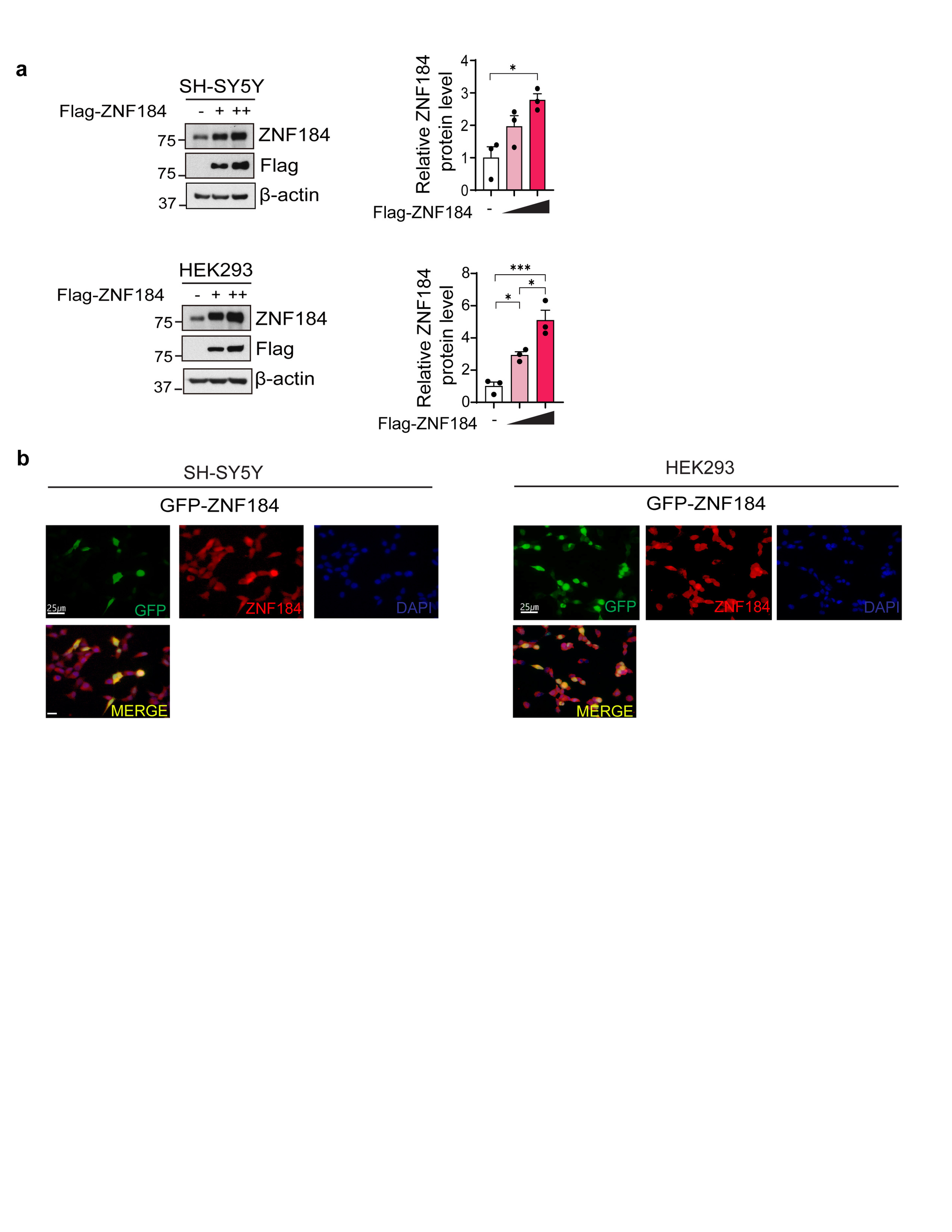

Supplement: S1 Fig — (a) Western blot analysis comparing endogenous and exogenous ZNF184 expression levels in HEK293 and SH-SY5Y cells transfected with Flag-ZNF184. (b) Immunostaining of GFP-ZNF184-transfected HEK293 and SH-SY5Y cells. GFP fluorescence (green) and anti-ZNF184 antibody staining (red) confirm robust expression of GFP-ZNF184 in both cell lines. Nuclei were counterstained with DAPI (blue). Scale bar: 25 um. (TIF) [file pone.0323279.s001.tif]

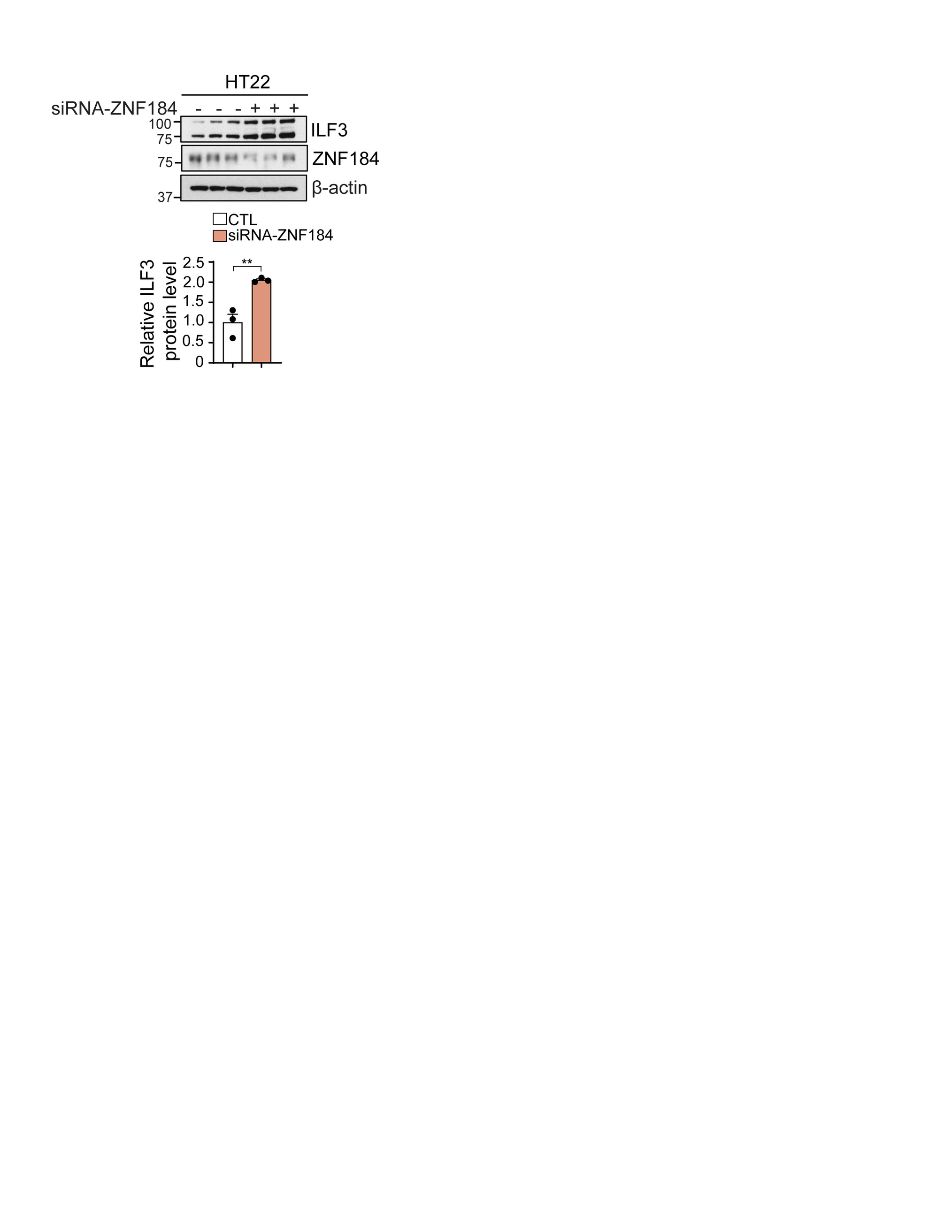

Supplement: S2 Fig — Quantification of the immunoblot normalized to β-actin. Statistical significance was assessed through a one-way ANOVA and unpaired t-test, with significance levels defined as follows: **p < 0.01. (TIF) [file pone.0323279.s002.tif]
