# Supplementary material for: Zinc finger protein 184 prevents α-synuclein preformed fibril-mediated neurodegeneration through the interleukin enhancer binding factor 3-microRNA-7 pathway
Source: PLoS One. 2025 May 7;20(5):e0323279. doi: 10.1371/journal.pone.0323279 (PMC12057860; doi:10.1371/journal.pone.0323279)
Supplement: S1 Table — (DOCX) [file pone.0323279.s003.docx]

**S1 Table.** Antibodies used in this study.

| Antibody | Dilution ratio | Source | Catalog number |
| --- | --- | --- | --- |
| Rabbit polyclonal anti-ZNF184 | 1:3000 | ProteinTech | 26100-1-AP |
| Rabbit polyclonal anti-ZNF184 | 1:1000 | Biorbyt | orb324804 |
| Rabbit polyclonal anti-NF90/ILF3 | 1:3000 | ProteinTech | 19887-1-AP |
| Rabbit polyclonal anti-Hsp90 | 1:3000 | Abcam | ab13495 |
| Rabbit monoclonal anti-Na^+^/K^+^ ATPase | 1:3000 | Thermo Scientific | MA542645 |
| Rabbit monoclonal anti-KDM1/LSD1 | 1:3000 | Abcam | ab129195 |
| Mouse monoclonal anti-GFP(B-2) | 1:3000 | Santa Cruz | sc-9996 |
| Mouse monoclonal anti-FLAG M2 (HRP conjugated) | 1:5000 | Sigma | A8592 |
| Rabbit polyclonal anti-TH | 1:3000 | Novus | NB300-109 |
| Mouse monoclonal anti-α-syn (phospho S129) | 1:3000 | BioLegend | MMS-5091 |
| Rabbit monoclonal anti-α-syn (phospho S129) | 1:3000 | Abcam | ab51253 |
| Mouse anti-α-syn | 1:1000 | BD Bioscience | BD-610787 |
| Rabbit polyclonal anti-PAR | 1:3000 | Trevigen | 4336-BPC-100 |
| HRP anti-β-actin [AC-15] | 1:5000 | Abcam | ab49900 |
| Goat anti-rabbit IgG (biotin-conjugated) | 1:1000 | Jackson | 111-065-045 |
| Goat anti-mouse IgG (HRP-conjugated) | 1:1000 | Abclon | Abc-5001-2 |
| Goat anti-rabbit IgG (HRP-conjugated) | 1:10000 | Bethyl | A120-101P |
| Donkey anti-mouse IgG H&L [Alexa Fluor 488] | 1:1000 | Abcam | ab150105 |
| Donkey anti-rabbit IgG H&L [Alexa Fluor 594] | 1:1000 | Abcam | ab150076 |
