# Supplementary material for: Zinc finger protein 184 prevents α-synuclein preformed fibril-mediated neurodegeneration through the interleukin enhancer binding factor 3-microRNA-7 pathway
Source: PLoS One. 2025 May 7;20(5):e0323279. doi: 10.1371/journal.pone.0323279 (PMC12057860; doi:10.1371/journal.pone.0323279)
Supplement: S2 Table — (DOCX) [file pone.0323279.s004.docx]

**S2 Table.** Primers used in this study.

| Target | Forward primer (5'→3') | Reverse primer (3'→5') | Experiment |
| --- | --- | --- | --- |
| human ZNF184 | TGTGCAGACTGGGAGACAAG | CAACAGACACTGCCAAACGA | RT-qPCR |
| mouse ZNF184 | TCAACGTTTATCCGTCACCA | CTGGTCATCCCTAGCTCAGC | RT-qPCR |
| human ILF3 | ACAGCAGGCTACAGTCAGTT | TTCTTCCCAGCGTGTTTTGG | RT-qPCR |
| human PFDN6 | GGGAGGCAGAAACTTGAAGC | TCCCTCTGTTGCTCTGACTG |  |
| human MDH1 | GAAGGGAAGGCATGGAGAGA | GGGATGGATGGAGCTGACTT |  |
| human RPL21 | CCCACAAGTGTTACCATGGC | GCGCTTTAGTTGAACCCAGG |  |
| human TMEM106C | GGCATCTGGTTTGGTGGTTT | CTGCCACCGTGTAGAAGTTG |  |
| human actin | AGAGCTACGAGCTGCCTGAC | AGCACTGTGTTGGCGTACAG |  |
| human ARPC2 | AAGGAAGGAGAGAACAGGGC | TGGCTAAAGAGGACCTGTGG |  |
| human SDCBP | GGTGGCTCCTGTAACTGGTA | GCTCCATCCTGCACAGTTTT |  |
| human ZNF184 binding motif | ACGCCTTAGCGAAACCAGA | CCAACCCAAATGGGAGGGAG | ChIP |
| MT1  (mutant 1) | TGCACGTGGTGCCCGCGTTAGTGTTTGCGTGCGTAGTCACGT | ACGTGACTACGCACGCAAACACTAACGCGGGCACCACGTGCA | Luciferase assay |
| MT2  (mutant 2) | GGGGGGGGCTGACGCCGTGGGCGTGAGT | ACTCACGCCCACGGCGTCAGCCCCCCCC |  |
